# Aerosols modification with H_2_O_2_ reduces airborne contamination by dental handpieces

**DOI:** 10.1080/20002297.2021.1881361

**Published:** 2021-01-29

**Authors:** Andrei Cristian Ionescu, Eugenio Brambilla, Lamberto Manzoli, Giovanna Orsini, Valentina Gentili, Roberta Rizzo

**Affiliations:** a Oral Microbiology and Biomaterials Laboratory, Department of Biomedical, Surgical and Dental Sciences, University of Milan, Milan, Italy; b Department of Medical Sciences, University of Ferrara, Ferrara, Italy; c Department of Clinical Sciences and Stomatology, Polytechnic University of Marche, Ancona, Italy; d Department of Chemical and Pharmaceutical Sciences, University of Ferrara, Ferrara, Italy

**Keywords:** SARS-CoV-2, disease transmission, aerosols, coronavirus infections, dental equipment, hydrogen peroxide

## Abstract

**Objective**: We designed an *in vitro* study to evaluate the efficiency of an 0.5 vol% hydrogen peroxide-based spray in reducing Coronavirus 229E spread during a conventional dental procedure.

**Methods**: A class III cabinet-like chamber was custom-built, using phantoms for both patient and operator. A suspension of HCoV-229E in artificial saliva having a similar viral load to SARS-CoV-2 asymptomatic patients was inoculated inside the patient’s phantom mouth. A 10 s-lasting dental procedure was performed using an aerosol-generating air-turbine, with or without high-volume evacuation (HVE). The effect of 0.5 vol% H_2_O_2_ cooling spray in reducing viral loads was tested. Viral presence on the operator phantom was assessed by Real-Time quantitative PCR on the mask’s outer surface, on the phantom’s forehead, and inside its mouth.

**Results**: When the H_2_O_2_ cooling spray was used, as compared to the conventional spray, viral loads were significantly lower on all tested sites, falling below the detection limit. Viral loads did not significantly change in any tested site when HVE was used.

**Conclusion**: The use of 0.5 vol% H_2_O_2_ cooling spray by dental handpieces drastically reduced the possibility of coronaviruses spread during aerosol-generating dental procedures. This strategy deserves further consideration among the preventive measures to be adopted during the SARS-CoV-2 pandemic.

## Introduction

The Coronavirus disease 2019 (COVID-19) pandemic has had a very high impact on medical and dental care since its outbreak [1,2]. Procedures for infection control and personal protective equipment (PPE) in the dental setting have been updated and, in some cases, profoundly changed to face this new emerging infective agent, based on minimal knowledge about the present disease [3-5]. There was an initial tendency to translate data from already-known coronaviruses and other airborne-spread diseases [6,7].

During dental procedures, the aerosol generation has long been regarded as a high-risk factor for airborne transmission of several diseases [8–10]. Pathogenic bacterial species, such as *Mycobacterium tuberculosis, Legionella pneumophila*, and *Staphylococcus* spp. as well as viruses (HIV, HBV, HCV, HSV, influenza virus, and rhinovirus) can all be spread by dental aerosols, reaching most surfaces in the dental operatory [10–12]. The cooling spray of the dental handpieces is the primary source of spatter and aerosol [13–15], and some studies have demonstrated the surprisingly wide distribution that such aerosols can have, showing contamination of virtually any surface of the dental operatory [16,17].

As the salivary glands are important reservoirs of coronavirus infection [18] in both symptomatic and asymptomatic subjects [19], the risk of infection spreading in the dental operatory by aerosol generation is very high [8,20]. Finally, early data suggest that SARS-CoV-2 can remain viable and infectious in aerosol for hours and on surfaces for days [21].

Since the literature on the influence of aerosol-generating procedures on the spreading of infective agents is limited [22], strategies are required to reduce the SARS-CoV-2 infection risk by dental aerosols and spatter. The main measures that have been proposed so far are to mitigate aerosol spreading range from the use of dental dams to high-vacuum air suction coupled with several nozzle designs [23], or using dental handpieces without water spray cooling, or even limiting dental treatments to those achievable without using dental handpieces [24].

Several disinfecting agents have been suggested and were efficacious to prevent surface contamination by coronaviruses [25–27]. In particular, a 0.5 vol% hydrogen peroxide solution was proposed as an ideal agent that could be used as water spray for dental handpieces since, in addition to its biocidal activity, it shows low toxicity for patient’s tissues [28], as well as a low degradation potential on the dental equipment [29]. Such a possibility would allow for a safe return to routine care, possibly avoiding current limitations consequent to aerosol spreading containment.

In the present study, we evaluated the potential efficacy in reducing the risk of human coronavirus transmission of an 0.5 vol% hydrogen peroxide solution into the cooling water spray, comparing the viral loads measured on the dental operator during simulated dental procedures, with or without H_2_O_2_ addition.

## Materials and Methods

### Experimental dental setting

**Figure 1.**
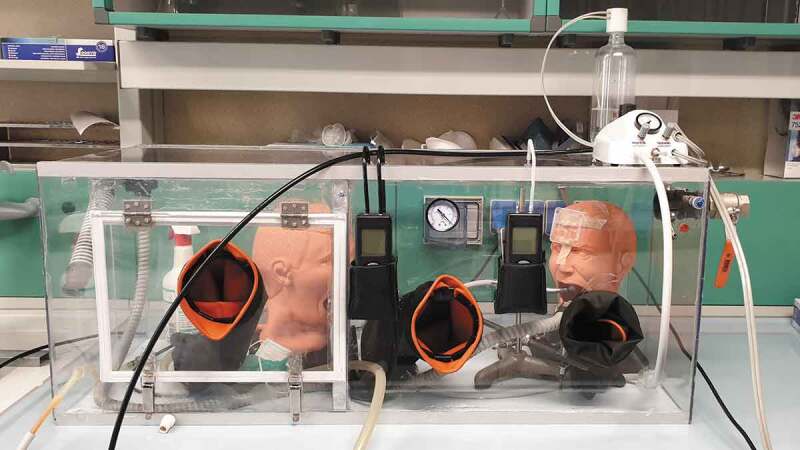
Setup of the custom-built Class III-like air-tight glove box with chamber pressure control. Three accesses for gloves are shown on the front panel, one created at the center of the door. Between the glove apertures, two digital manometers and a backup analog manometer measured the negative pressure inside the chamber (right digital manometer and analog manometer) and the differential pressure inside the mouth of the operator phantom when a mask covered its mouth and nose (left digital manometer). On the upper right corner of the chamber, the two air leak valves are visible for pressure control. The control apparatus operating the air turbine is located on the right part of the upper panel, having attached the pressurized water tank to generate the air-water cooling spray for the turbine handpiece. The chamber is still to be connected to an oil-free air compressor, HVE line, and two low-vacuum pumps, here not shown

A polycarbonate pressure-tight chamber (100 cm x 40 cm x 40 cm) was custom-built (Figure 1). A total of three circular holes (10 cm in diameter) and a door (35 cm x 25 cm) were mounted on the front panel. Three 50 cm-long latex gloves were fitted to the circular apertures. The chamber was connected through air-tight tubing to two laboratory vacuum pumps, a dental high-vacuum system (HVE, flow rate: 1,700 l/min; maximum operating head: 280 mbar, TurboSmart 2 v, Cattani S.P.A., Parma, Italy), and a handpiece tubing providing a connection for an air turbine. Two digital manometers (measuring range: 200 ± 0.1 mbar) were fixed to the external part of the front panel. Two air leak valves were mounted to set a constant negative pressure of 15 mbar inside the chamber to avoid contamination of the surrounding area. The first valve allowed for fine-tuning of the pressure while the other one (1–1/2” diameter) allowed to compensate for a higher air intake due to the HVE operation. When HVE was not operating, the second valve was closed, and a non-return valve on the HVE line ensured that back-contamination was prevented. All holes, the panel, and the tubing connections were sealed to make the chamber air-tight and bear constant negative operating pressure.

**Figure 2.**
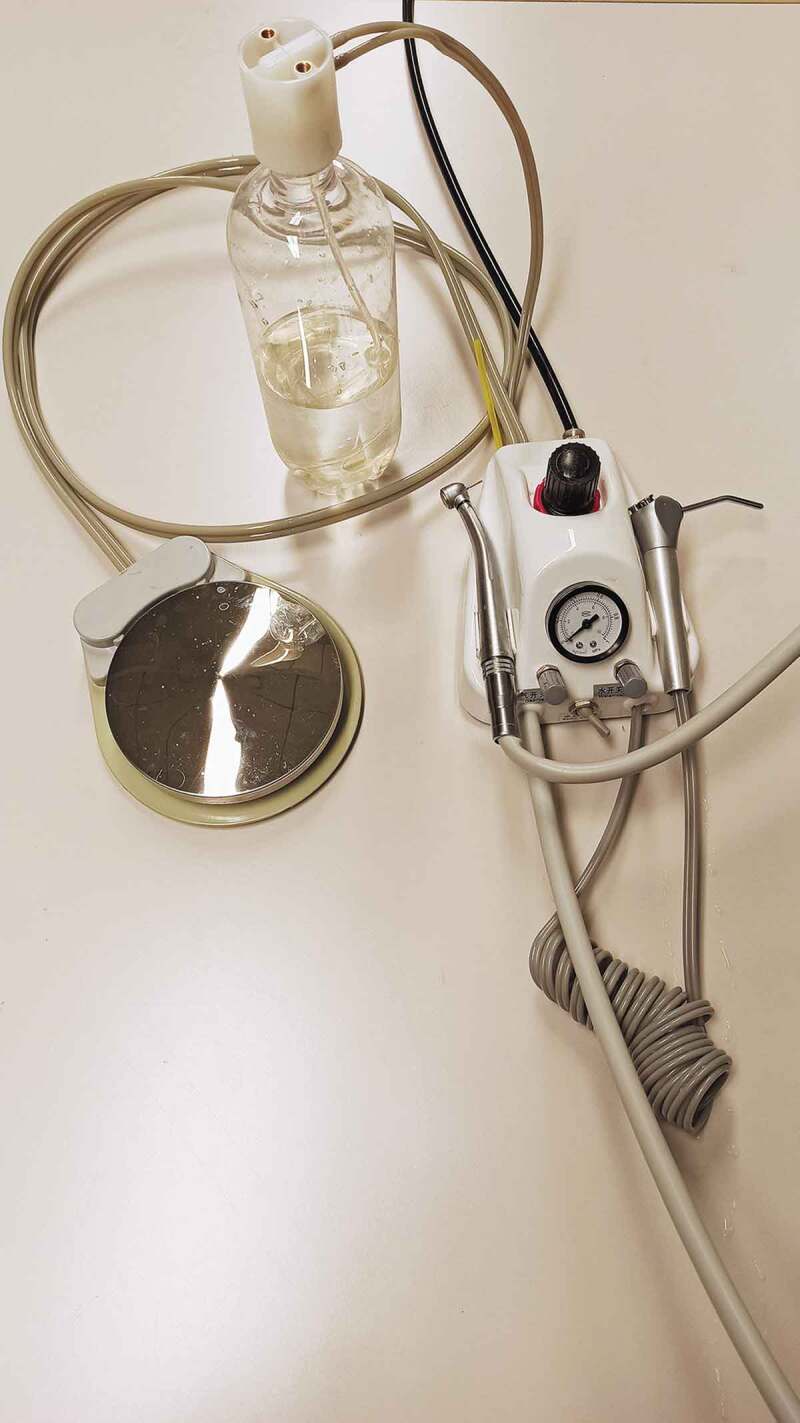
Presentation of the air turbine and control apparatus used in the present study. The pressurized water tank used to generate the air-water cooling spray for the turbine handpiece is seen on the upper part. The handpiece cord was afterwards inserted inside the chamber and sealed, for the turbine to operate inside the patient phantom

A dental portable turbine unit (Zhengzhou Kongsin Trading Co. Ltd, Zhengzhou City, Henan, China) was connected to a dental air compressor and used to operate the air turbine handpiece located inside the chamber (Bora, Bien-Air Dental SA, Bienne, Switzerland). The latter was equipped with a cylindrical diamond bur (835KR.314.016, Komet Italia Srl, Milan, Italy). The air pressure was set to 3.3 atm, and the speed was 320,000 r.p.m. The system was provided with a 1,000 ml water tank for the air-water spray (Figure 2).

**Figure 3.**
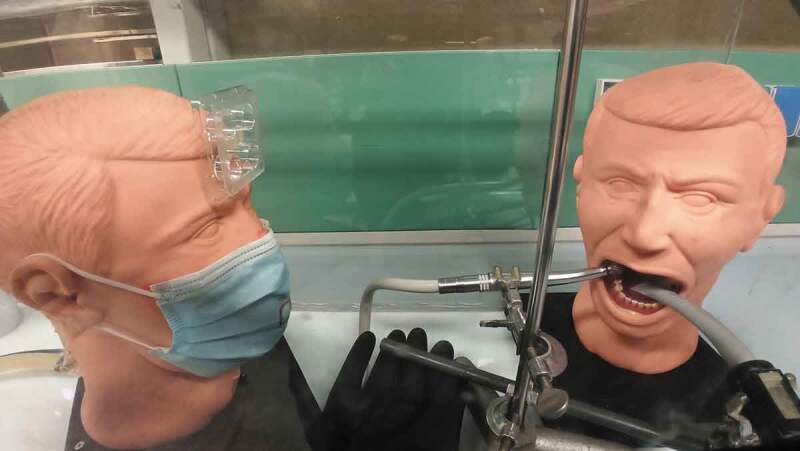
The two phantoms inside the chamber are shown. On the right the patient phantom is situated, having the air-turbine and HVE tip fixed in the same position throughout all experimental runs, as if operated by a right-handed dentist and dental assistant. On the left, the operator phantom equipped for the first experimental run, with a surgical mask and the first target fixed with double-sided adhesive tape on its forehead. The external area of the mask to be assessed for viral contamination can be seen marked in red. In all runs, the chamber space between operator and patient was left free of materials and protruding gloves to allow for an even aerosol spread, similarily to clinical conditions

Two phantom heads (operator and patient) were fixed to custom-made holders in a vertical position inside the chamber at a conventional working distance of 25 cm (Figure 3). The patient’s phantom was equipped with resin teeth (Columbia Dentoform Corp., Long Island City, NYC, USA). The air turbine and the HVE were oriented to simulate a right-handed dentist’s position during the preparation of the occlusal surface of the lower right first molar. A universal laboratory holder was used to hold the air turbine and the HVE in the same position for the experiments’ whole duration. The air turbine was positioned 2 mm away from the tooth surface and oriented towards the inner part of the dental arch, while the HVE tip was positioned on the opposite side of the tooth. The distance between the HVE tip and the turbine tip was 2 cm.

The operator phantom was sealed, making it pressure-tight, except for the mouth opening. The phantom was connected to a low-vacuum pump to simulate the air-flow during inspiration, and to one of the manometers. The operator phantom was provided with a custom made tray (70 mm x 70 mm) to accommodate a 4-well plate (Nunc IVF, Merck, Darmstadt, Germany) inside its mouth. A site was identified and marked on the phantom’s forehead to position another 4-well plate using double-sided adhesive tape. In this way, data about viral surface contamination were gathered on three sites, namely the operator’s forehead, mask surface, and the interior of the operator’s mouth (behind the mask). Finally, a sprayer containing absolute ethanol to be used during operation procedures was inserted on the chamber’s side opposite the phantoms.

### Preparation of hydrogen peroxide solution

An 0.5 vol% H_2_O_2_ solution was obtained using distilled water and a stock 50 vol% H_2_O_2_ solution immediately before the experiments.

### Preparation of viral solutions

For safety issues, a coronavirus (Human coronavirus 229E, ATCC® VR-740) was used as a biological tracer instead of SARS-CoV-2. A suspension of HCoV-229E with a viral load of 6.03 ± 0.04 log_10_ gene copies/ml that resembles SARS-CoV-2 saliva levels of asymptomatic infected subjects [30,31] was prepared in an artificial saliva solution. The artificial saliva simulated the average electrolyte composition of human whole saliva and was prepared from 0.1 L of 150 mM KHCO_3_, 0.1 L of 100 mM NaCl, 0.1 L of 25 mM K_2_HPO_4_, 0.1 L of24 mM Na_2_HPO_4_, 0.1 L of 15 mM CaCl_2_, 0.1 L of 1.5 mM MgCl_2_, and 0.006 L of 25 mM citric acid. The volume was made up to 1 L, a total of 2.5 g mucin (type II, porcine gastric) was added, and the pH was adjusted to 7.0 by pipetting 4 M NaOH or 4 M HCl solutions under vigorous stirring [32]. Cryo-vials, each containing 1 ml of stock viral suspension, were prepared and frozen at −80°C. On the day of testing, stock suspensions were thawed and stored in an ice bath. Experiments were performed in triplicate.

### Operation procedures

All personnel operating the chamber wore protective equipment, including gloves, FFP3 respirators and face shields, and disposable gowns. Before starting each experimental run, two 4-well target plates were placed in their corresponding locations (inside the operator phantom’s mouth and on its forehead) with their lid closed. A surgical mask was positioned on the operator phantom, taking care to adapt it over the nose and the mouth openings and removing the 4-well lid just before positioning of the mask. A 1.9 cm^2^ square was marked in a central position on the outer part of each mask. A sterile, leakproof plastic bag was positioned in the chamber to collect specimens after the experimental run. A flask of sterile PBS solution, a micropipette with its sterile tips, sterile scissors, and sterile Eppendorf tubes were also inserted into the chamber. Then, one vial containing the viral suspension was positioned on a stand inside the chamber. Vacuum pumps and HVE were turned on to reach operating pressure conditions inside the chamber. After that, all procedures inside the chamber were performed using air-tight gloves. The micropipette was used to transfer the viral solution (1 ml) on the bottom of the lower arch inside the patient phantom, mimicking saliva drain from the submandibular glands. The air turbine was then operated for a total of 10 s to generate an aerosol containing the viral particles. After that, HVE was turned off, and a 60 s time were allowed for aerosol dispersion. Then, the mask was removed, and the forehead and the mouth plates were covered with their lid. The mask and the two plates were positioned in the plastic bag and hermetically sealed. The bag was marked with the corresponding code of the experimental run. The sprayer was then used on all chamber surfaces. A total of 60 s was allowed for ethanol disinfection and evaporation, then the HVE was turned on again to remove residual ethanol completely. The chamber’s negative pressure was equalized to the atmospheric pressure; the door was opened to remove the specimen-containing bag and discard the equipment used during the run safely.

The experimental conditions tested were the followings:

1. HVE, air turbine cooled by tap water spray;
2. No HVE, air turbine cooled by tap water spray;
3. No HVE, air turbine cooled by 0.5 vol% H_2_O_2_ water spray, waiting for a 30 s contact time before collecting the targets.
4. No HVE, air turbine cooled by 0.5 vol% H_2_O_2_ water spray, waiting for a 60 s contact time before collecting the targets.

During experimental runs #3 and #4, at the end of the contact time (30 s or 60 s, respectively), samples from the external layer of the surgical masks of the same size as the target’s wells (1.9 cm^2^) were cut out from the mask using the sterile scissors. Samples were placed in a sterile Eppendorf vial containing 500 µl of PBS. Each well of the targets was washed with 500 µl of PBS; then, the solution was transferred to an Eppendorf vial. The tubes were placed inside the plastic bag, then disinfection procedures of the chamber were performed as previously described. Each experimental run was performed four times, and the results averaged.

### Determination of viral loads on the target surfaces

At the end of each experimental run, the target-containing bag was immediately transferred to the virological laboratory in the next room. The wells of each target were washed with 500 μl PBS; the solution was collected in sterile Eppendorf vials and stored at −80°C until analysis. Samples with the same surface area as the target’s wells (1.9 cm^2^) were cut from the mask’s external layer, inserted in Eppendorf vials containing 500 μl PBS, and stored as previously described. HCoV-229E presence on targets was assessed using Real-Time quantitative PCR (qPCR). RNA extraction was performed using the Purelink viral RNA/DNA kit (ThermoFisher, Milan, Italy). A total of 500 μl of viral suspension was used, and the elution was performed with 10 μl elution buffer. RNA was retrotranscribed with Superscript VILO cDNA synthesis kit (ThermoFisher) and amplified with an HCoV-229E specific qPCR gene assay (Vi06439671_s1, Catalog number: 4,331,182, ThermoFisher).

### Statistical analyses

All analyses were performed using SAS software (JMP 14.0, SAS Institute, Cary, NC, USA). qPCR data were analyzed after log-transformation to approach a normal distribution, which was verified using Shapiro-Wilk’s test. The limit of detection for qPCR in an error-free environment, where only sampling noise contributes to the variation, was calculated to be three viral copies at a 95% confidence interval [33]. Considering the noise due to extraction and reverse transcription, the limit of detection was conservatively determined as four viral copies, according to the methodology proposed by Forootan and coworkers [34]. Data were expressed in Log_10_viral copies/cm^2^. Homogeneity of variances was checked using Levène’s test, and two way ANOVA model was used considering the site (forehead, mask tissue, and mouth) and the experimental setting (HVE, H_2_O_2_ spray, and contact time) as fixed factors. Tukey’s test was used to evaluate significant differences between groups. The significance level was set at a two-sided p < 0.05.

## Results

**Figure 4.**
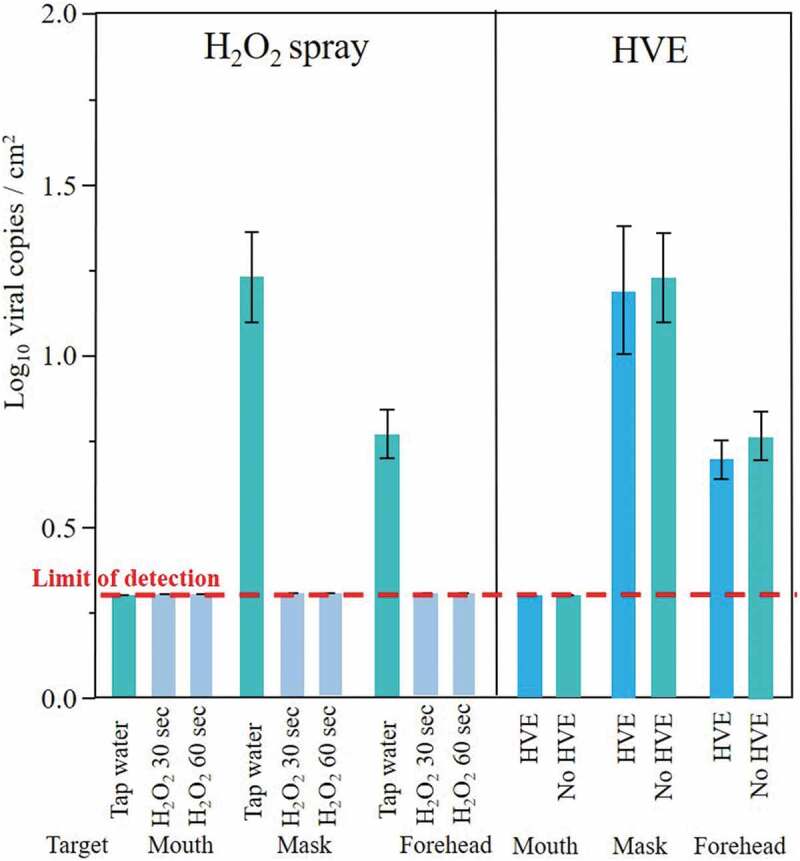
Graph showing the results of the experimental runs (mean Log_10_ viral copies/cm^2^ ± 1SE). Results are divided by the aims of the study, that is to compare the efficacy of high-vacuum suction (HVAC) and the addition of 0.5 vol% hydrogen peroxide to the cooling spray of the dental handpiece (H_2_O_2_ spray) in mitigating coronavirus spread by aerosol. Experimental runs using HVE were performed using surgical mask, while runs using H_2_O_2_ spray were performed using surgical mask and turning off the HVE

The detected viral loads were relatively low compared to the artificial saliva content. Expectedly, the highest viral loads were found over the masks’ external surfaces (Table 1, Figure 4). The viral RNA was found on the forehead targets during runs using tap water spray with significantly lower loads than the external surfaces of the masks (p < 0.001). When the H_2_O_2_ cooling spray was used, the viral load was under the detection limit on all tested sites, including mask surfaces and operator’s forehead, with no significant differences between the tested times (p > 0.10, Figure 4). Inside the operator’s mouth, the viral load was under the detection limit in all tested conditions. No significant differences were found for any tested site when HVE was used (p > 0.10).

**Table 1.** Results (mean±SD) of the log-transformed viral copies per square centimeter for each experimental run and target. Different letters indicate significant differences between groups: e.g. the values with the letter ‘a’ are significantly different from those with the letters ‘b’ or ‘c’ (Tukey’s test, p < 0.05)

Exp. run	Target	Configuration	Suction	Log_10_viral copies/cm^2^	
1	Mouth	Surgical mask	HVE	0.317 ± 0 *	c
2	Mouth	Surgical mask	No HVE	0.317 ± 0 *	c
3	Mouth	Surgical mask + H_2_O_2_ 30 sec	No HVE	0.317 ± 0 *	c
4	Mouth	Surgical mask + H_2_O_2_ 60 sec	No HVE	0.317 ± 0 *	c
1	Mask	Surgical mask	HVE	1.214 ± 0.538	a
2	Mask	Surgical mask	No HVE	1.249 ± 0.372	a
3	Mask	Surgical mask + H_2_O_2_ 30 sec	No HVE	0.317 ± 0 *	c
4	Mask	Surgical mask + H_2_O_2_ 60 sec	No HVE	0.317 ± 0 *	c
1	Forehead	Surgical mask	HVE	0.711 ± 0.164	b
2	Forehead	Surgical mask	No HVE	0.783 ± 0.212	b
3	Forehead	Surgical mask + H_2_O_2_ 30 sec	No HVE	0.317 ± 0 *	c
4	Forehead	Surgical mask + H_2_O_2_ 60 sec	No HVE	0.317 ± 0 *	c

* Under the detection limit, determined to be equal to 4 viral copies/sample.

## Discussion

The cooling spray generated by dental handpieces represents the primary source of spatter and aerosol in dentistry [8,9], being able to spread droplets and contaminate virtually any surface of the dental operatory [16,17]. From this point of view, this study aimed to test the efficacy of a modified water cooling spray for dental handpieces in preventing the spread of Coronavirus, simulating in a pressure-tight air chamber an aerosol-generating procedure commonly used in the dental operatory.

Several disinfectants have been proven effective against Coronaviruses. According to Kampf, high-concentration alcohols, aldehydes, povidone-iodine, sodium hypochlorite, and hydrogen peroxide were all able to reduce coronavirus infectivity [25,26]. Of note, hydrogen peroxide was shown to exhibit virucidal activity, starting from a low concentration (0.5 vol%) and 60 s contact time. Due to its extensive use in applications, such as in the food industry, where its decomposition into non-toxic by-products is essential [35], H_2_O_2_ was the ideal candidate to be tested for the modification of the dental handpiece cooling spray. All other disinfectants were excluded due to toxicity concerns, fire or explosive hazard, or corrosion issues regarding dental handpieces and waterlines. Also, it must be noted that the majority of modern dental units are provided with an internal sanitization circuit that is conceived to be used with H_2_O_2_ starting from 0.5 vol%, at the end of the working day, for biofilm removal [29].

To the best of our knowledge, this is the first study to test a hydrogen peroxide solution as a permanent modifier of the water cooling spray to a dental unit’s handpieces. In this simulation, the addition of an 0.5 vol% H_2_O_2_ reduced the viral loads spread by aerosols below the detection limits in all measured sites, even with the shortest contact time that was tested (30 s). The main potential explanation is an intense degradation activity exerted by hydrogen peroxide on viral RNA. This activity is due to an excess generation of reactive oxygen species, which exhibits a strong denaturing effect mainly directed against nucleic acids [35]. H_2_O_2_ has also demonstrated degradation activity of proteins and lipidic membranes, suggesting the main inactivation mechanisms against Coronaviruses responsible for its substantial virucidal activity [35,36]. Besides, vaporizing or fogging hydrogen peroxide confers it a higher activity than its liquid form [36], in agreement with the effects observed in the present study using a relatively low concentration (0.5 vol%) and a short contact time (30 seconds) of aerosolized H_2_O_2_. Other disinfectants such as chlorhexidine, cinnamon extract, or povidone-iodine have been tested as promising antibacterial coolants, especially for ultrasonic scaling procedures [37]. However, their virucidal activity, when aerosolized by dental handpieces, has never been evaluated.

HVE has been proposed among the strategies to mitigate aerosol and spatter propagation in the dental operatory. A recent Cochrane review by Nagraj and coworkers concluded that the use of an HVE reduces bacterial contamination in aerosols less than one foot (about 30 cm) from a patient’s mouth but not at longer distances [37]. Furthermore, Ravenel and coworkers recently tested the efficacy of several dry-field isolation techniques, including HVE alone, against spatter and aerosol generation [23]. They found that HVE alone significantly reduced spatter, yet it did not reduce aerosol detection compared to the negative control (ambient room values). Our results, showing no difference in the amount of aerosolized viral copies recovered with or without the use of HVE at a conventional working distance of 25 cm, agree with these findings, showing that HVE may not be an effective way to reduce aerosols at such a distance range. We must nevertheless highlight some differences in these studies’ setup, including the distance between the bur and the tip of the HVE (2 cm in our study vs. 4 cm in Ravenel et al.) and the tip of the HVE.

In the present study, a phantom head simulating the dental operator was connected to a vacuum pump that replicated inspiration by continuous air intake through the surgical masks. The results indicate that, when dental control procedures were performed without the use of hydrogen peroxide, viral loads on the masks’ outer surfaces were significantly higher than those on the phantom’s forehead, in spite of a similar distance to the infection source. This interesting result can be explained considering that a vacuum pump was connected to the operator phantom to simulate inspiration by continuous air intake through the tested PPEs. The air-flow through the mask allowed the aerosol particles carrying the viral load to concentrate on its surfaces. In a study by Prospero and coworkers, the level of bacterial contamination on several surfaces of the dental operatory was assessed and compared [38]. They found that healthcare workers’ masks were significantly more contaminated than were all other surfaces. Another study evaluated the contamination by respiratory viruses on the outer surfaces of medical masks used by hospital healthcare workers, finding contamination on a median of 10% of the masks [39]. The authors concluded that respiratory pathogens on the outer surface of the used medical masks might result in self-contamination. As the results of these studies and the present one outline, extreme care should be taken when disposing of surgical masks as they may concentrate any airborne pathogen on their surface and be a significant source of self-contamination for dental operators. This possibility might be highly reduced by using hydrogen peroxide-containing water cooling for dental handpieces, as the generated aerosol may help disinfect the outer surface of the masks.

This study, not unlike any other, has strengths and limitations that must be discussed. A negative pressure chamber was custom-built according to a Class III cabinet’s specifications to accurately replicate the clinical operative conditions in a standardized way. Operator’s and patient’s phantom position, distance, and the handpiece setup were chosen to simulate a clinical situation where a conventional procedure was performed. The tested viral solutions were prepared using artificial saliva and inoculated into the patient phantom’s oral cavity immediately before each experimental run. This procedure was aimed to approximate as closely as possible the characteristics of aerosol spreading in the clinical setting.

Although we did not use SARS-CoV-2 for safety issues, the chosen infective agent (Human Coronavirus 229E) has very similar characteristics (dimensions and structure), accurately reproducing its spread by the aerosols generated in the dental setting, using a viral concentration most commonly found in the saliva of infected, asymptomatic subjects [30,31].

Most protocols adopted in the dental setting starting from the COVID-19 first wave aimed to intercept symptomatic subjects before being admitted to dental care. These protocols included double triage procedures, and body temperature measurements and oximetry [7]. Such protocols are, by all means, unable to intercept asymptomatic subjects. It is now known that the spread of the disease by the latter poses the main risk, especially during procedures involving aerosol generation.

The study’s main limitations consist of using a fixed distance between the phantoms, performing a single dental procedure, and the duration of the aerosol-spreading dental procedure that lasted only 10 s. The duration was selected to allow complete dispersion of the viral inoculum through aerosol and spatters during each experimental run. An increase in operating time, such as the one necessary for tooth crown preparation, would have needed much higher amounts of viral inoculum that would have been difficult to produce and manage.

The contamination of the whole operatory, for safety issues, could not be tested using the present setup, therefore the influence of many parameters such as patient-operator distance, orientation of the patient, and working site, on viral contamination still has to be ascertained. It is known that airborne spread of diseases in the dental setting is quite an issue [8], with virtually any surface of the clinic environment being contaminated when, as in the present setting, an air turbine is operated [16]. However, dental health-care providers stand in the area of maximum contamination, therefore a reduction of viral contamination of the operator remains a good indicator that the tested procedures were effective.

Finally, the use of eye protection is strongly suggested to reduce the risk of SARS-CoV-2 contamination [4]. Given the similar distance of the forehead and eyes from the infection source, it might be inferred that the eyes had been exposed to similar amounts of contamination, further confirming the need for eye protection of dental health-care providers.

Further studies assessing extended periods and various distances are also needed to evaluate the safety of the 0.5 vol% H_2_O_2_ solution for patients and operators, to map the reduction in viral contamination across the operatory and the potential deteriorating activity of such solution on handpieces and dental chair waterlines.

## Conclusions

An 0.5 vol% of H_2_O_2_ added to the water spray of dental handpieces drastically reduced the possibility of coronaviruses spread during aerosol-generating dental procedures. These results, needing confirmation, indicate this procedure as one of the main strategies to limit SARS-CoV-2 transmission in the dental setting.
